# Lifestyle, medication and socio-demographic determinants of mental and physical health-related quality of life in people with multiple sclerosis

**DOI:** 10.1186/s12883-016-0763-4

**Published:** 2016-11-22

**Authors:** George A Jelinek, Alysha M De Livera, Claudia H Marck, Chelsea R Brown, Sandra L Neate, Keryn L Taylor, Tracey J Weiland

**Affiliations:** Neuroepidemiology Unit, Melbourne School of Population and Global Health, The University of Melbourne, Melbourne, VIC 3010 Australia

**Keywords:** Multiple sclerosis, Quality of life, Determinants, Epidemiology

## Abstract

**Background:**

Health-related quality of life (QOL) is a key outcome for people with multiple sclerosis (MS). While modifiable lifestyle factors, like smoking, physical activity and vitamin D, have strong associations with development and progression of MS, few studies have examined such associations with QOL.

**Methods:**

Using patient-reported data from 2312 people with MS from 54 countries, regression models explored associations of socio-demographic, therapeutic and lifestyle factors with QOL, using the Multiple Sclerosis Quality of Life-54 (MSQOL-54).

**Results:**

Participants were on average 45.6 years old, 82.4% women, mostly partnered (74.1%), with a university degree (59.5%). Controlling for socio-demographic factors and disability, factors associated with better physical health composite (PHC) (on a 100 point scale) were: moderate and high physical activity compared to low (5.9 [95% confidence interval: 4.2, 7.6] and 9.9 [CI: 8.1, 11.6] points higher score respectively); non-smoking compared to current smoking (4.6 points [CI: 2.4, 6.7]); better diet (per 10 points on the 100 point Diet Habits Questionnaire scale (DHQ) 1.6 points [CI: 1.0, 2.2] points); normal body mass index (BMI) versus overweight or obese (2.1 points [CI: 0.4, 3.7] and 2.4 points [CI: 0.5, 4.3]); fewer comorbidities (4.4 points [CI: 3.9, 4.9]); and not taking a disease-modifying drug (DMD) (2.1 points [CI: 0.7, 3.4]).

Better mental health composite (MHC) determinants were: moderate and high physical activity compared to low (4.0 points [CI: 2.0, 6.0] and 5.7 points [CI: 3.5, 8.0]); non-smoking compared to current (6.7 points [CI: 4.1, 9.3]); better diet (2.8 points [CI: 1.9, 3.5]); normal BMI versus overweight or obese (3.1 points [CI: 1.1, 5.1] and 3.5 points [CI: 1.3, 5.7]); meditating regularly (2.2 points [CI: 0.2, 4.2]); and no DMD use (2.9 points [CI: 1.3, 4.6]).

**Conclusions:**

While causality cannot be concluded from cross-sectional data, the associations between modifiable lifestyle factors and QOL suggest significant potential for secondary prevention of the known deterioration of QOL for people with MS through lifestyle risk factor modification.

**Electronic supplementary material:**

The online version of this article (doi:10.1186/s12883-016-0763-4) contains supplementary material, which is available to authorized users.

## Background

Multiple sclerosis (MS) is increasingly recognized as a disease with modifiable lifestyle components influencing development and progression [1] that significantly impairs health-related quality of life (QOL) [2, 3]. QOL has been an important component of health status since the early 1980s [4], initially describing generic physical and mental health status in chronic disease, soon adapted to suit particular illnesses [5], including MS [6]. QOL is a key outcome for people with MS [7] that needs to be measured in clinical studies [8, 9], may predict the course of disability [10–12], and should be used more widely by clinicians [10], potentially forming the main objective of management [13].

While studies have highlighted the importance of psycho-social issues in determining QOL in MS [13, 14], particularly depression and fatigue [15, 16], there has been little research on associations of lifestyle and socio-demographic factors with physical and mental QOL. Potentially determining these associations may allow clinicians to provide evidence-based advice to people with MS on the relative importance of modifiable lifestyle risk factors that should be prioritized for optimal QOL. Many of these factors are associated with QOL and premature death in the general population [17], but there has been conflicting advice regarding diet, stress reduction and other lifestyle behaviours for the MS population. Additionally, currently available disease-modifying drugs (DMDs) have been shown to reduce relapse rate and in some cases slow progression to disability, but there has been conflicting evidence about their effects on QOL, varying from medication to medication, largely related to the issue of side effects [9, 18].

Our previous research has separately examined associations of a number of individual lifestyle risk factors with QOL in a large international sample of people with MS, allowing detailed analysis of the relationship between QOL and diet [19] including specific dietary components [20], latitude and vitamin D supplementation [21], physical activity [22], alcohol consumption and cigarette smoking [23], and body mass index (BMI) and number of comorbidities [24], as well as DMD use [25]. These studies did not however allow comparison of the relative magnitude of these associations while mutually adjusting for other modifiable lifestyle factors, medication use and potentially related socio-demographic factors. Such evidence will pave the way for research both to verify and trial these lifestyle determinants in suitably designed intervention studies, and to potentially design and refine treatment programs to integrate medication and lifestyle risk factor modification into secondary preventive approaches.

In this study, using patient-reported data from the Health Outcomes and Lifestyle In a Sample of people with MS (HOLISM) Study [26], we examined a full suite of mutually adjusted lifestyle and socio-demographic factors in this large international sample of people with MS, allowing evaluation of the relative magnitude of associations between lifestyle, medication and socio-demographic factors, and physical and mental QOL.

## Methods

### Participants and data collection

The HOLISM study has been described in detail elsewhere [26]. In brief, participants with definite or possible MS were approached through online forums and websites to undertake a cross-sectional survey using Survey Monkey®. Respondents providing consent completed an online questionnaire incorporating validated tools where possible. Demographic factors included age, gender, marital status, educational status, and occupational status using researcher-devised items. Disability was measured with Patient Determined Disease Steps (PDDS) [27] and co-morbidities with The Self-Administered Comorbidity Questionnaire (SCQ) [28]. Body mass index (BMI) was calculated according to the World Health Organisation (WHO) criteria (<20 = underweight, 20-25 = normal weight, 25-30 = overweight and >30 = obese). Dietary Habits Questionnaire (DHQ) [29], designed to measure diet according to cardiovascular rehabilitation guidelines, was modified slightly to assess diet on a scale of 0-100. Social support was measured with the Single Item Measure of Social Support [30].

Level of regular physical activity (low, moderate and high) was assessed using the International Physical Activity Questionnaire (IPAQ) [31]. The IPAQ categories briefly represented the following: high active meant vigorous-intensity activity on at least 3 days, or 7 days of walking, or moderate or vigorous activity; moderately active meant 3 or more days of vigorous activity of at least 20 min per day; or 5 or more days of moderate or higher intensity activity or walking of at least 30 min per day; and low active meant no or some activity. Omega-3 and vitamin D supplementation (low [average daily dose <5000 IU], high [average daily dose ≥5000 IU]), meditation practice, level of alcohol consumption (low [<15 g/week], moderate [up to 30 g/day for women and 45 g/day for men], or high) and current smoking status were assessed using researcher-devised items. Current use of DMDs was assessed using researcher-devised questions [25].

### Exclusion criteria

The initial dataset consisted of 3132 people with definite or possible MS. Of these, 663 who did not have confirmed MS and 157 not providing adequate information were excluded, leaving 2312 individuals for analyses.

### Measuring QOL

The Multiple Sclerosis Quality of Life (MSQOL-54) Instrument [6] was used as a quantitative measure of physical health-related QOL (Physical Health Composite Score or PHC) and mental health-related QOL (Mental Health Composite Score or MHC). The MSQOL-54, developed from the 36 item short-form health survey (SF-36) to measure QOL in people with MS, is sound psychometrically, in widespread use and available in many languages [10].

### Statistical analysis

Data were analysed using Stata, V12 (StataCorp, College Station, Texas, USA) and R (R Foundation for Statistical Computing, Vienna, Austria). Categorical, continuous, and discrete data were presented as frequency (percentages), mean (standard deviation) and median (25th–75th percentile) respectively.

#### Main analysis

To explore associations of modifiable and non-modifiable risk factors with QOL, using HOLISM variables and our subject-matter knowledge [19–25], we devised a causal outline to develop clinically meaningful multivariable regression models separately for PHC and MHC, with mutual adjustment for lifestyle risk factors, DMD use and socio-demographics. Complete case analysis was restricted to participants with available data.

### Sensitivity analyses

#### Multiple imputation

Analyses were undertaken using multiple imputation to account for missing data. Fully Conditional Specification [32] was used with a single imputation model consisting of the variables in Table 1. To improve performance of the imputation model [33], some of these variables were used as auxiliary variables (variables not included in the analysis model, but either correlated with the missing variables or associated with missingness of the variables). Fifty multiply imputed datasets were created, and results from these 50 datasets combined using Rubin’s rules [33].

**Table 1:** Characteristics of outcome, modifiable and stable variables

Variables		Value (*n* = 2312)
Outcome variables		
*MSQOL*	Physical health composite^a^	59.0 (21.5)
	Mental health composite^a^	66.6 (21.4)
Modifiable variables		
*Latitude (degrees)* ^*a*^		41.0 (8.7)
*Diet (DHQ total)* ^*a*^		78.9 (12.1)
*Current smoker* ^*b*^		268/2289 (11.7)
*Alcohol consumption* ^*b*^	Low	1398/2275 (61.5)
	Moderate	858/2275 (37.7)
	High	19/2275 (0.8)
*Vitamin D supplementation* ^*b*^	Low	1747/2194 (79.6)
	High	447/2194 (20.4)
*Exercise (IPAQ)* ^*b*^	Low	922/2232 (41.3)
	Moderate	707/2232 (31.7)
	High	603/2232 (27.0)
*Omega 3 supplementation* ^*b*^	None	806/2198 (36.7)
	Flaxseed only	204/2198 (9.3)
	Fish oil only	793/2198 (36.0)
	Both	395/2198 (18.0)
*Meditation* ^*b*^	Never or less than once a week	1568/2243 (69.9)
	Once a week or more	675/2243 (30.1)
*BMI* ^*b*^	Obese	439/2284 (19.2)
	Overweight	521/2284 (22.8)
	Normal	1230/2284 (53.9)
	Underweight	94/2284 (4.1)
*Number of support people* ^*b*^	One or none	596/2224 (26.8)
	Two or more	1628/2224 (73.2)
Socio-demographics		
*Age* ^*a*^		45.6 (10.6)
*Gender* ^*b*^	Male	403/2288 (17.6)
*Employment* ^*b*^	Employed full or part time	1323/2256 (55.3)
	Student or stay at home carer	244/2256 (10.2)
	Unemployed	175/2256 (7.8)
	Retired due to age or disability	602/2256 (26.7)
*Marital status* ^*b*^	Partnered	1691/2281 (74.1)
	Single	327/2281 (14.4)
	Separated	263/2281 (11.5)
*Education level* ^*b*^	Didn’t complete high school	46/2303 (2.0)
	Completed high school/trade	886/2303 (38.5)
	Completed bachelor/higher degree	1371/2303 (59.5)
*Number of comorbidities* ^*c*^		1 (0,2)
*Disability scale* ^*b*^	Normal to some	1267/2299 (55.1)
	Gait/cane	792/2299 (34.5)
	Major mobility	240/2299 (10.4)
*Years since diagnosis* ^*c*^		6 (3,12)
*Country of origin*	Australasia	808/2312 (34.9)
	Europe	606/2312 (26.2)
	North America	841/2312 (36.4)
	Other	57/2312 (2.5)
*DMD use*	Yes	1096/2312 (51.4)

^a^Values are mean (SD), ^b^Values are number (%), ^c^Values are median (25th–75th percentile), Denominators vary reflecting number of respondents completing each item

#### Re-weighted PHC

We also performed a re-weighted analysis in order to explore the effect of the missing sexual health component. While all variables had a relatively low percentage of missing values (<5%), PHC was missing in 12.6% of cases, mostly (84%) due to participants not disclosing sexual health data. To explore the effect of inclusion of individuals whose sexual health component was excluded in calculating PHC, we carried out a secondary analysis using multivariable regression, after calculating a re-weighted PHC that excluded the sexual health function.

## Results

### Sample characteristics

Characteristics of outcome, modifiable and non-modifiable variables are summarised in Table 1. Our study sample of people with MS from 54 countries, predominantly North America, aged on average in their mid-forties, with an unusually high ratio (over 4:1) of women, was well educated with healthy lifestyle habits. This likely reflects selection bias in sampling relatively health-literate people with MS engaged in web-based groups seeking information about health. Their diet was healthy in terms of complying with a cardiovascular risk-reduction diet. There was a low proportion of smokers with few heavy alcohol drinkers, a high proportion supplemented with vitamin D and omega 3s, nearly a third meditated regularly, and the majority engaged in moderate to high physical activity. A little over half were currently using a DMD.

### Determinants of QOL

Table 2 presents associations between modifiable factors and PHC and MHC using complete case analysis. Noteworthy associations are bolded. While we have previously examined the associations of each of the variables separately with QOL [19–25], these regression models allowed us to determine associations of each modifiable factor with QOL in the context of all other stable and modifiable factors. While there is no definite agreement on the magnitude of MSQOL-54 score difference that constitutes a clinically relevant difference, as it is derived from the SF-36, and a 5 point difference on this scale has previously been considered to be clinically significant [34], this level of difference was considered a convenient approximation.

**Table 2:** Associations between modifiable factors and PHC and MHC obtained from multivariable regression models using complete case analyses

Variables		Physical Health Composite^a^Coefficient (95% CI)	*p*-value	Mental Health Composite^b^Coefficient (95% CI)	*p*-value
*Latitude (per 10 degrees)*		**-1.3 (-2.0, -0.5)**	**0.001**	-0.8 (-1.9, 0.1)	0.1
*BMI*	Normal	reference			
	Underweight	0.2 (-3.2, 3.7)	0.9	1.5 (-2.8, 5.9)	0.5
	Overweight	**-2.1 (-3.7, -0.4)**	**0.02**	**-3.1 (-5.1, -1.1)**	**0.002**
	Obese	**-2.4 (-4.3, -0.5)**	**0.005**	**-3.5 (-5.7, -1.3)**	**0.002**
*Alcohol consumption*	Low	reference			
	Moderate	0.3 (-7.8, 8.3)	0.9	0.9 (-7.8, 9.6)	0.8
	High	-2.1- (-10.1, 5.9)	0.6	-1.8 (-10.6, 6.8)	0.7
*Number of comorbidities*		**-4.4 (-4.9, -3.9)**	**<0.001**		
*Diet: DHQ total (per 10 points)*		**1.6 (1.0, 2.2)**	**<0.001**	**2.8 (1.9, 3.5)**	**<0.001**
*Current smoker*		**-4.6 (-6.7, -2.4)**	**<0.001**	**-6.7 (-9.3, -4.1)**	**<0.001**
*Vitamin D supplementation*	Low	reference			
	High	1.4 (-0.2, 3.1)	0.1	-0.04 (-2.1, 2.0)	0.9
*Omega 3 supplementation*	None	reference			
	Flaxseed only	**2.5 (0.0, 5.1)**	**0.05**	2.1 (-1.0, 5.2)	0.2
	Fish oil only	1.0 (-0.6, 2.6)	0.2	1.0 (-0.9, 3.0)	0.3
	Both flaxseed and fish oil	2.0 (-0.1, 4.1)	0.06	0.4 (-2.2, 3.0)	0.8
*Exercise (IPAQ)*	Low	reference			
	Moderate	**5.9 (4.2, 7.6)**	**<0.001**	**4.0 (2.0, 6.0)**	**0.001**
	High	**9.9 (8.1, 11.6)**	**<0.001**	**5.7 (3.5, 8.0)**	**<0.001**
*Meditation frequency*	Never or < once a week	reference			
	Once a week or more	-.5 (-2.0, 1.0)	0.7	**2.2 (0.2, 4.2)**	**0.03**
*Number of support people*	None or one	reference			
	Two or more	**3.1 (1.6, 4.6)**	**<0.001**	**6.7 (4.9, 8.5)**	**<0.001**
*Marital status*	Single			reference	
	Partnered			**4.7 (2.4, 7.0)**	**<0.001**
	Separated			1.8 (-1.4, 5.0)	0.3
*Employment status*	Unemployed			reference	
	Student or stay at home carer			**7.0 (3.2, 10.9)**	**<0.001**
	Employed full or part time			**9.4 (6.3, 12.5)**	**<0.001**
	Retired due to age or disability			**4.8 (1.5, 8.1)**	**0.005**
*Education level*	Didn’t complete high school	reference			
	High school and/or trade	3.6 (-1.1, 8.3)	0.14		
	Bachelor or higher degree	**4.9 (0.2, 9.6)**	**0.04**		
*DMD use*	Yes	**-2.1 (-3.4, -0.7)**	**0.003**	**-2.9 (-4.6, -1.3)**	**0.002**

^a,b^Models were adjusted for gender and disability level (age was excluded as in this sample age was collinear with disability level). ^a^The complete case analysis consisted of 76% of individuals. ^b^The complete case analysis consisted of 82% of the individuals. Number of comorbidities which included measures of the MHC including depression was not included in this model

### Physical health composite

Those with moderate and high, compared with low, physical activity scored approximately 6 points and 10 points higher respectively on the PHC; not smoking versus currently smoking was associated with 4-5 points better PHC; improved diet of around 30 points on the 100 point DHQ was associated with a clinically relevant 5 points higher PHC; and flaxseed oil supplementation compared to none was associated with around a 2-3 point increment, although significance was marginal and disappeared on multiple imputation.

Of relatively stable but also potentially modifiable factors, number of comorbidities had a strong association, with one less comorbidity associated with 4-5 points higher PHC, along with having two or more support people at around 3 points higher QOL compared to one or none. In keeping with recent research showing higher relapse rate [21, 35] and disability [35] with increasing latitude, our data showed 1-2 points lower PHC for every 10 degrees in latitude further from the equator, an association that, while potentially modifiable, may not be of clinically relevant magnitude. Those with normal BMI compared with overweight or obesity had a 2-3 point better PHC. Higher education was associated with around 5 points better QOL. We found no significant association between alcohol intake and QOL, despite having previously found a positive association when examining this factor individually [23]. Currently taking a DMD was associated with around a 2 point lower PHC.

### Mental health composite

Smoking was significantly associated, with non-smokers having 6-7 points better MHC than smokers. Improved diet of around 20 points was associated with a clinically relevant 5-6 points higher MHC; moderate compared with low exercise was associated with a 4 point increment, and high versus low around 6 points. Meditating once a week or more was associated with around 2 points better QOL compared with not.

Of relatively stable socio-demographic factors, employment was associated with 9-10 points higher MHC for those employed, and 5-7 points for retirees and students compared to those unemployed, and 6-7 points higher score for those with two or more support people. Those who were partnered had around 5 points better QOL than those not. Normal BMI was associated with 3-4 points better MHC compared with overweight or obesity. Taking a DMD was associated with around a 3 points worse MHC.

### Sensitivity analyses

The characteristics of individuals with PHC missing compared to individuals with the PHC were explored (Additional file 1: Table S1). Those with PHC missing were older, female and either single or separated. Variables age, marital status, employment status and MHC were used as auxiliary variables for analysing PHC, and variables age, education status, number of comorbidities and PHC for MHC. Hence, a single imputation model consisting of all variables was used for multiple imputation within Fully Conditional Specification.

### Multiple imputation

Associations between modifiable factors and PHC and MHC obtained using multiple imputation to account for missing values, were essentially consistent with complete case analysis (Additional file 1: Table S2).

### Re-weighted PHC

Results from the multivariable model that included individuals with missing sexual health data were broadly consistent with multiple imputation and complete case analysis (Additional file 1: Table S3).

## Discussion

Our study, with participants exhibiting a full range of exposures to lifestyle factors, and around half not taking a DMD, provided an important opportunity to investigate the relationship of lifestyles ranging from poor to very healthy, controlling for DMD use, with QOL outcomes in MS. The cohort had better than average health status, reflected in body weight, with BMI considerably lower than current Western norms, and only one in ten of the sample with major mobility issues. In turn, this may account for the relatively high physical and mental QOL of the group overall, comparable to a recent Canadian cohort of people with ‘benign’ MS [36].

While causality cannot be concluded from our cross-sectional study, the demonstrated associations of modifiable lifestyle factors with QOL highlight the potential for secondary prevention of the known deterioration of QOL for people with MS through lifestyle risk factor modification. Of factors readily modifiable, cessation of cigarette smoking offers enormous potential benefits to both mental and physical health status of people with MS in addition to its general health benefits. Similarly, while reverse causality may have contributed to the magnitude of effect, a potential minimized by controlling for disability, regular moderate or vigorous exercise warrants recommendation where appropriate in view of the strength of the association with mental and particularly physical QOL, and its known beneficial effect on general health.

Traditionally, people with MS have been advised that dietary change has no proven benefit; in contrast, our data show significantly better QOL for those eating a better diet in line with current cardiovascular recommendations that promote a wholefood diet with reductions in saturated fat intake and increased consumption of fruit, vegetables and fish [37]. Our previous research focusing solely on diet showed these particular individual dietary factors had the strongest associations with the health of people with MS [19]. In the current study, around 20 points increase on the 100 point DHQ scale was associated with clinically relevant higher mental health QOL and 30 point increase with higher physical QOL. Twenty to 30 points improvement appears quite feasible in terms of potential dietary change, representing a change from a relatively typical modern Western diet to a cardiovascular-health diet.

Our study both supported previously described associations of relatively stable socio-demographic factors with physical and mental QOL of people with MS [38], and revealed new independent associations of significant magnitude of several important modifiable lifestyle determinants. This supports further epidemiological research in validating these factors, and clinical research targeting these lifestyle factors in suitable trials. It has already been shown for example that smoking cessation after MS diagnosis results in a highly significant delay to reaching a progressive phase of the disease of around eight years [39]; presumably this translates to benefits for QOL, and this warrants further study.

While the adoption of healthy lifestyle behaviours may be good for the health status of a person with MS, many would assume that these changes, such as increasing exercise, quitting smoking and improving diet, are difficult to initiate and maintain. Our findings that both mental and physical QOL are better with healthier lifestyle behaviours may provide motivation for people with MS adopting healthy lifestyles and clinicians to recommend such changes. People simply feel better.

QOL is a complex construct. There is an inter-play between psychosocial factors such as depression and social isolation, stress, and locus of control or self-efficacy, as well as immune regulation, disease progression and QOL for people with MS [40]. Plausible biological pathways explain how modifiable lifestyle factors may potentially influence many parts of this equation both directly and indirectly, affecting not only immune function and disease activity directly [41], but also mood, coping mechanisms and sense of control over the illness.

Adopting healthy behaviours may be indirectly associated with QOL, as an externally oriented locus of control, with a feeling of being unable to influence health outcomes, predicts worse disability but also depression and fatigue [42], both determinants of QOL in their own right, and both strongly associated with the lifestyle factors examined here [43, 44]. Having healthy behaviours may be associated with positive orientation of locus of control, with attendant benefits in motivation to access health information and better adherence to treatment [42]. Where studied, the great majority of people with MS have an external locus of control, a proportion that increases with disease duration [45].

Our study shows that smoking cessation, healthy diet and regular exercise appear to be key targets for lifestyle recommendations in people with MS, whether or not they take a DMD. While DMD use was associated with worse QOL both physically and mentally, the decrease was probably not of clinical significance. Nonetheless, as some of the medications approved for MS have significant side effects and may well worsen QOL, our data suggest lifestyle modification may also be of benefit in ameliorating such negative effects, with the potential for adoption of healthy lifestyles to improve tolerance of the DMDs for those who take them.

Formal study of the QOL of people with MS who adopt these behaviours will provide the necessary evidence for their adoption in secondary preventive programs; however each of these factors is independently associated with improved QOL, with incrementally better QOL the healthier these behaviours, that is a dose-response association, lending weight to the likelihood of a causal association and encouragement to those willing to embrace them, whether or not they take DMDs. Weight loss also requires further study with respect to causality and in intervention studies as a secondary preventive measure in MS, with our study demonstrating significant associations of obesity with worse QOL. Structured programs to improve and increase social support and employment participation should also be studied in light of the demonstrated associations with QOL.

Primary care physicians may find it helpful to indicate to their patients with MS the magnitude of these differences in QOL for people with MS with healthy compared with unhealthy lifestyles. For example, our data indicate that comparing two people with MS with similar socio-demographics and disability, one who doesn’t smoke, has a normal BMI and eats a wholefood diet low in saturated fat with frequent seafood scoring 90 points on the DHQ, with a high level of physical activity (such as swimming and running four days of the week for 45 min to an hour), without other illnesses, would score 30 points higher in physical QOL and 24 points higher in mental QOL compared with someone who smokes, is overweight, eats a typical Western diet with few vegetables or fruit scoring 60 on the DHQ, has a low level of physical activity, and has back pain and high blood pressure, commonly seen in people with such lifestyles. QOL differences of such magnitude are of great clinical significance.

### Limitations

Our data were self-reported, and therefore accuracy cannot be verified. Each of the validated tools used has inherent limitations; we also used a number of researcher-devised questions not previously validated. The highly educated and relatively healthy nature of our sample may make our findings less generalizable. As this was an observational cross-sectional study, we cannot be clear about causality; the contribution of reverse causality may have been significant for some factors, such as physical activity, although it appears implausible for many of the associations noted, such as cigarette smoking and diet. Temporality may be a limitation, in that QOL was measured over the four weeks prior to the survey, whereas some of the other measures used different timeframes. Planned longitudinal follow up of the participants should provide evidence about temporality. Strengths of the study include the large sample representing over 50 countries and all clinical types of MS, enabling wide generalizability, and the absence of commercial interests, reducing potential for this bias.

## Conclusion

Cigarette smoking, diet and exercise are associated with QOL for people with MS, both physically and mentally, with a dose-response effect for healthier diet and more exercise associated with increasingly better QOL, suggesting a causal relationship. Other lifestyle factors showed positive associations with QOL to a lesser extent, and DMD use a negative but probably clinically insignificant association. More research is required to clarify the magnitude and importance of these associations, and to incorporate lifestyle interventions into robust clinical trials. However, for optimal health status, lifestyle advice for people with MS appears warranted.

## Additional file

Additional file 1: Table S1. Characteristics of the individuals with physical health composite missing and non-missing. **Table S2.** Associations between modifiable factors and physical and mental health composites obtained from multivariable regression models using multiple imputation. **Table S3.** Associations between modifiable factors and the recalculated physical health composite obtained from the multivariable regression model using complete case analysis. (DOCX 32 kb)

## Abbreviations

DMDs: Disease-modifying drugs
HOLISM: Health outcomes and lifestyle in a sample of people with MS
IPAQ: International physical activity questionnaire
MHC: Mental health composite score
MS: Multiple sclerosis
MSQOL-54: Multiple Sclerosis Quality of Life-54﻿ instrument
PDDS: Patient Determined Disease Steps
PHC: Physical health composite score
QOL: Health-related quality of life
SCQ: Self-Administered Comorbidity Questionnaire
SF-36: 36 item short-form health survey
WHO: World Health Organisation
