# Supplementary material for: Lifestyle, medication and socio-demographic determinants of mental and physical health-related quality of life in people with multiple sclerosis
Source: BMC Neurol. 2016 Nov 22;16:235. doi: 10.1186/s12883-016-0763-4 (PMC5120469; doi:10.1186/s12883-016-0763-4)
Supplement: Additional file 1: Table S1. — Characteristics of the individuals with physical health composite missing and non-missing. Table S2. Associations between modifiable factors and physical and mental health composites obtained from multivariable regression models using multiple imputation. Table S3. Associations between modifiable factors and the recalculated physical health composite obtained from the multivariable regression model using complete case analysis. (DOCX 32 kb) [file 12883_2016_763_MOESM1_ESM.docx]

Supplementary Table 1

Characteristics of the individuals with physical health composite missing and non-missing

|  | **Physical health composite** | |
| --- | --- | --- |
| **Variable** | **missing (n=291)** | **non-missing (n= 2021)** |
| Disability scale |  |  |
| Normal to some | 45.45% | 56.48% |
| gait/cane | 39.16% | 33.78% |
| major mobility | 15.38% | 9.74% |
| Mental health composite [mean (sd)] | 65.46 (21.1) | 66.77 (21.43) |
| Latitude (degrees) [mean (sd)] | 41.71 (9.12) | 40.91 (8.64) |
| DHQ [mean (sd)] | 79.28 (10.87) | 78.83 (12.3) |
| Smoker |  |  |
| yes | 90.14% | 88.03% |
| Alcohol |  |  |
| low | 66.31% | %60.77 |
| moderate | 32.62% | %38.43 |
| high | 1.08% | %0.8 |
| Vitamin D |  |  |
| low | 84.36% | 78.95% |
| high | 15.64% | 21.05% |
| IPAQ |  |  |
| low | 20.22% | 27.98% |
| moderate | 29.96% | 31.92% |
| high | 49.82% | 40.10% |
| Omega 3 supplementation |  |  |
| none | 34.67% | 36.95% |
| flaxseed only | 10.22% | 9.15% |
| fish oil only | 35.40% | 36.17% |
| both | 19.71% | 17.72% |
| Meditation |  |  |
| never | 67.26% | 70.29% |
| once a week | 32.74% | 29.71% |
| BMI |  |  |
| normal | 54.04% | 53.83% |
| underweight | 5.61% | 3.9% |
| overweight | 17.89% | 19.41% |
| obese | 22.46% | 22.86% |
| Age [mean (sd)] | 50.08 (11.5) | 44.96 (10.29) |
| Gender |  |  |
| male | 6.5% | 19.14% |
| Employment |  |  |
| unemployed | 9.86% | 7.45% |
| student or stay at home carer | 7.39% | 10.65% |
| employed full or part time | 48.94% | 56.24% |
| retired due to age or disability | 33.80% | 25.66% |
| Marital status |  |  |
| single | 29.23% | 12.22% |
| married | 47.54% | 77.92% |
| separated | 23.24% | 9.86% |
| Number of comorbidities [median (25^th^-75^th^ percentile)] | 1 (0,2) | 1 (0,2) |
| Education level |  |  |
| didn’t complete high school | 1.38% | 2.09% |
| completed high school and/or trade | 38.62% | 38.45% |
| completed bachelor or higher degree | 60.00% | 59.46% |
| Number of support people |  |  |
| one or none | 33.45% | 25.84% |
| two or more | 66.55% | 74.16% |
| DMD use | 38.5% | 50.6% |

Supplementary Table 2

Associations between modifiable factors and physical and mental health composites obtained from multivariable regression models using multiple imputation

| **Variable** | **Physical Health Composite^1^** | | **Mental Health Composite^2^** | |
| --- | --- | --- | --- | --- |
|  | **Coefficient** | **p-value** | **Coefficient** | **p-value** |
|  | **(95% CI)** |  | **(95% CI)** |  |
| Latitude (per 10 degrees) | **-1.46(-2.16,-0.75)** | **<0.001** | **-0.86(-1.78,0.05)** | 0.1 |
| BMI |  |  |  |  |
| normal |  |  |  |  |
| underweight | -0.7(-3.84,2.44) | 0.66 | **1.55(-2.55,5.65)** | 0.459 |
| overweight | **-1.95(-3.47,-0.42)** | **0.01** | **-3.13(-5.15,-1.11)** | **0.002** |
| Obese | **-2.33(-4.06,-0.61)** | **0.01** | **-3.51(-5.73,-1.29)** | **0.002** |
| Alcohol consumption |  |  |  |  |
| low |  |  |  |  |
| moderate | -3.41(-10.15,3.33) | 0.32 | 0.89(-7.82,9.6) | 0.8 |
| high | -5.5(-12.18,1.18) | 0.11 | -1.88(-10.56,6.8) | 0.7 |
| Number of comorbidities | **-4.34(-4.79,-3.89)** | **<0.001** |  |  |
| DHQ total | **0.17(0.1,0.23)** | **<0.001** | **0.28(0.2,0.35)** | **<0.001** |
| Current smoker |  |  |  |  |
| no |  |  |  |  |
| yes | **-4.87(-6.81,-2.93)** | **<0.001** | **-6.7(-9.27,-4.14)** | **<0.001** |
| Vitamin D supplementation |  |  |  |  |
| low |  |  |  |  |
| high | **1.63(0.07,3.2)** | **0.04** | -0.05(-2.11,2.02) | 0.9 |
| Omega 3 supplementation |  |  |  |  |
| none |  |  |  |  |
| flaxseed only | 2.27(-0.1,4.64) | 0.06 | 2.1(-1.03,5.23) | 0.2 |
| fish oil only | 0.7(-0.76,2.15) | 0.35 | 1.04(-0.93,3.02) | 0.3 |
| both flaxseed and fish oil | 1.19(-0.77,3.16) | 0.23 | 0.36(-2.24,2.96) | 0.8 |
| IPAQ |  |  |  |  |
| low |  |  |  |  |
| moderate | **5.93(4.36,7.5)** | **<0.001** | **4.02(2.01,6.02)** | **<0.001** |
| high | **9.26(7.62,10.89)** | **<0.001** | **5.75(3.53,7.96)** | **<0.001** |
| Number of support people |  |  |  |  |
| none or one |  |  |  |  |
| two or more | **3.26(1.87,4.64)** | **<0.001** | **6.73(4.91,8.55)** | **<0.001** |
| Meditation frequency |  |  |  |  |
| never or less than once a week |  |  |  |  |
| once a week or more | -0.29(-1.71,1.13) | 0.7 | **2.12(0.29,3.95)** | **0.02** |
| Marital status |  |  |  |  |
| single |  |  |  |  |
| married |  |  | **4.71(2.41,7.01)** | **<0.001** |
| separated |  |  | 1.81(-1.39,5.01) | 0.3 |
| Employment status |  |  |  |  |
| unemployed |  |  |  |  |
| student or stay at home carer |  |  | **7.04(3.21,10.86)** | **<0.001** |
| employed full or part time |  |  | **9.37(6.29,12.46)** | **<0.001** |
| retired due to age or disability |  |  | **4.78(1.48,8.08)** | **0.005** |

^1^ The variables age, marital status, employment status and the mental health composite were used as auxiliary variables in the multiple imputation model. Analysis model was adjusted for gender, education level, and disability level. Age and employment status were not included in the model as they were collinear with disability and education respectively. Interesting associations are shown in bold. The variables age, education status, number of comorbidities and the physical health composite were used as auxiliary variables in the imputation model. Analysis model was adjusted for gender and disability level. Age and education level were not included in the model as they were collinear with disability and employment status respectively. Number of comorbidities which included measures of the mental health composite was not included in this model. Interesting associations are shown in bold.

Supplementary Table 3

Associations between modifiable factors and the recalculated physical health composite obtained from the multivariable regression model using complete case analysis

| **Variable** | **Physical Health Composite^1^** | |
| --- | --- | --- |
|  | **Coefficient** | **p-value** |
|  | **(95% CI)** |  |
| Latitude (per 10 degrees) | **-1.6(-2.3,-0.8)** | **<0.001** |
| BMI |  |  |
| normal |  |  |
| underweight | -1.03(-4.21,2.15) | 0.53 |
| overweight | **-1.83(-3.43,-0.23)** | **0.03** |
| Obese | **-2.68(-4.49,-0.87)** | **<0.001** |
| Alcohol consumption |  |  |
| low |  |  |
| moderate | -1.37(-8.58,5.84) | 0.7 |
| high | -3.78(-10.97,3.41) | 0.3 |
| Number of comorbidities | **-4.33(-4.81,-3.85)** | **<0.001** |
| DHQ total | **0.16(0.09,0.22)** | **<0.001** |
| Current smoker |  |  |
| no |  |  |
| yes | **-5.04(-7.12,-2.96)** | **<0.001** |
| Vitamin D supplementation |  |  |
| low |  |  |
| high | **1.7(0.11,3.29)** | **0.04** |
| Omega 3 supplementation |  |  |
| none |  |  |
| flaxseed only | 2.43(-0.01,4.87) | 0.05 |
| fish oil only | 1.44(-0.1,2.97) | 0.07 |
| both flaxseed and fish oil | 2.06(0.07,4.04) | 0.04 |
| IPAQ |  |  |
| low |  |  |
| moderate | **6(4.42,7.59)** | **<0.001** |
| high | **9.36(7.67,11.06)** | **<0.001** |
| Number of support people |  |  |
| none or one |  |  |
| two or more | **2.99(1.55,4.42)** | **<0.001** |
| Meditation frequency |  |  |
| never or less than once a week |  |  |
| once a week or more | -0.58(-2.04,0.88) | 0.9 |

^1^The complete case analysis consisted of 85% of individuals. Model adjusted for gender, education level, and disability level. Age and employment status were not included in the model as they were collinear with disability and education respectively. Interesting associations are shown in bold.
